# A randomized controlled trial: the effect of inulin on weight management and ectopic fat in subjects with prediabetes

**DOI:** 10.1186/s12986-015-0033-2

**Published:** 2015-10-24

**Authors:** Nicola D. Guess, Anne Dornhorst, Nick Oliver, Jimmy D. Bell, E. Louise Thomas, Gary S. Frost

**Affiliations:** Nutrition and Dietetic Research Group, Imperial College, London, Hammersmith Hospital, London, W12 0NN UK; Division of Diabetes, Endocrinology and Metabolism, Imperial College NHS Trust, London, UK; Department of Life Sciences, Faculty of Science and Technology, University of Westminster, London, W1W 6UW UK; Division of Diabetes, Endocrinology and Metabolism, 6th Floor Commonwealth Building, Faculty of Medicine, Imperial College Hammersmith Campus, Du Cane Road, London, W12 ONN UK

**Keywords:** Diabetes prevention, Diabetes risk, Weight management, Intrahepatocellular lipid, Intramyocellular lipid, Fibre, Carbohydrate, Appetite

## Abstract

**Background:**

Fat infiltration of the liver, muscle and pancreas is associated with insulin resistance and risk of diabetes. Weight loss reduces ectopic fat deposition and risk of diabetes, but is difficult to sustain to due to compensatory increases in appetite. Fermentable carbohydrates have been shown to decrease appetite and food intake, and promote weight loss in overweight subjects. In animal studies, fermentable carbohydrate reduces ectopic fat independent of weight loss. We aimed to investigate the effect of the fermentable carbohydrate inulin on weight maintenance, appetite and ectopic fat in subjects with prediabetes.

**Methods:**

Forty-four subjects with prediabetes were randomized to 18 weeks’ inulin or cellulose supplementation. During weeks 1–9 (weight loss phase) all subjects had four visits with a dietitian to guide them towards a 5 % weight loss. During weeks 10–18 (weight maintenance phase) subjects continued taking their assigned supplementation and were asked to maintain the weight they had lost but were offered no further support. All subjects attended study sessions at baseline, 9 and 18 weeks for measurement of weight; assessment of adipose tissue and ectopic fat content by magnetic resonance imaging and magnetic resonance spectroscopy; glucose, insulin and GLP-1 levels following a meal tolerance test; and appetite by *ad libitum* meal test and visual analogue scales.

**Results:**

Both groups lost approximately 5 % of their body weight by week nine (−5.3 ± 0.1 % vs −4.3 ± 0.4 %, *p* = 0.13, but the inulin group lost significantly more weight between 9 and 18 weeks (−2.3 ± 0.5 % vs −0.6 ± 0.4 %, *p* = 0.012). Subjects taking inulin had lower hepatic (*p* = 0.02) and soleus muscle (*p* < 0.05) fat content at 18 weeks compared to control even after controlling for weight loss and consumed less at the *ad libitum* meal test (*p* = 0.027). Fasting glucose significantly decreased at week nine only (*p* = 0.005), insulin concentrations did not change, and there was a significant increase in GLP-1 in the cellulose group at 9 and 18 weeks (*p* < 0.03, *p* < 0.00001).

**Conclusion:**

Inulin may have a two-pronged effect on the risk of diabetes by 1) promoting weight loss 2) reducing intrahepatocellular and intramyocellular lipid in people with prediabetes independent of weight loss.

**Trial registration:**

Clinical trial number: NCT01841073.

**Electronic supplementary material:**

The online version of this article (doi:10.1186/s12986-015-0033-2) contains supplementary material, which is available to authorized users.

## Background

Lifestyle interventions can significantly reduce the risk of developing type 2 diabetes (T2DM) [1] with weight loss being the primary mediator of the reduction in risk [2]. However, outside of a labour-intensive clinical trial setting, long-term weight loss is notoriously difficult to achieve [3, 4].

One explanation for the rarity of successful weight loss maintenance is that an energy deficit and loss of body fat are both associated with increases in appetite and food intake [5, 6]. Therefore, interventions such as glucagon-like peptide-1 (GLP-1) agonists aimed at decreasing or ameliorating such changes in appetite help promote long-term weight loss maintenance [7]. Non-digestible carbohydrate (dietary fibre) has long been linked to reduced food intake [8], and fermentable carbohydrates (FCHO) may be particularly effective [8]. This class of carbohydrate passes undigested and unabsorbed from the upper gastrointestinal tract to the colon where bacterial fermentation produces short chain fatty acids (SCFA) that can stimulate GLP-1, and regulate other appetite hormones [9]. Furthermore, as a natural dietary compound, FCHOs could represent a widely applicable public health intervention. By promoting a natural reduction in appetite, less frequent clinical support may needed. In a year-long study in overweight adolescents, there was a significantly lower rise in body mass index (BMI) in the inulin group, without receiving input from health professionals [10].

A key mediator of the beneficial effect of weight loss on insulin sensitivity is the loss of ectopic fat [11], which is highly correlated to insulin resistance and T2DM [11–13]. Intriguingly, FCHO has been shown to reduce ectopic fat in animal studies independent of weight loss [14]. This raises the possibility that this natural dietary carbohydrate may not only enhance weight loss efforts, but may also promote loss of this metabolically-deleterious fat in humans, even after accounting for weight loss. In healthy and insulin-resistant subjects, FCHO has been shown to improve insulin sensitivity [15, 16], but it is not clear whether a reduction in ectopic fat plays a role. No previous study has examined the effect of a dietary intervention on ectopic fat in subjects with prediabetes.

Here we report the effect of 30 g/day (following a 4-week dose escalation period) inulin supplementation taken alongside a 9-week weight loss program and a 9-week weight maintenance period. We hypothesise the inulin will result in greater weight loss maintenance at the 18-week follow-up in subjects with prediabetes, and will promote a reduction in ectopic fat and insulin resistance independent of weight loss assessed by using the gold-standard magnetic resonance imaging (MRI) and magnetic resonance spectroscopy (MRS) techniques.

### Volunteers

Subjects with known prediabetes or high risk factors for prediabetes were identified from local GPs registers. Informed consent was obtained prior to the study. The protocols were approved by the North West 1 Research Ethics Committee (registration number: 10/H0717/32) (Clinical trial number: NCT01841073) and conformed to the Declaration of Helsinki. An oral glucose tolerance test (OGTT) was performed to clarify glycaemic status [17]. Inclusion criteria were age ≥ 18 years, BMI of 25–35 kg/m^2^, prediabetes (impaired fasting glucose (IFG) and/or impaired glucose tolerance (IGT)) using the American Diabetes Association criteria [17] and a stable body weight for 3 months prior to the study. Exclusion criteria were normal glucose tolerance, T2DM, gastrointestinal disorders, pregnancy or breastfeeding, prescribed medication that affects appetite or glucose homeostasis and consumption of prebiotic products or antibiotic use within 3 months of the study start date. Volunteers were randomised by BMI and gender using a random-number table, with an allocation ratio of 1:1.

### Experimental design

**Fig. 1:**
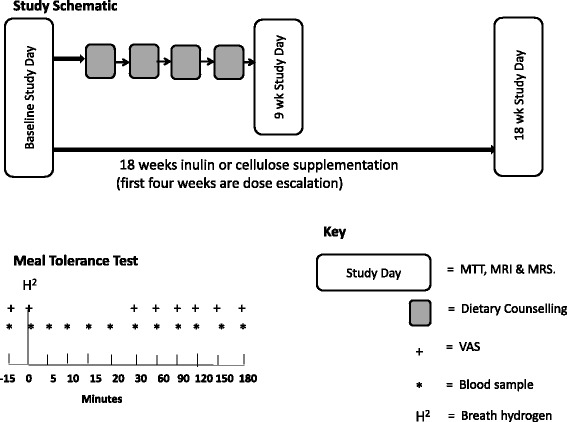
Schematic showing study outline, including the timings of blood samples, VAS and breath hydrogen measure during the MTT. H^2^: breath hydrogen measure; MTT: meal tolerance test; MRS: magnetic resonance imaging; MRS: magnetic resonance spectroscopy; VAS: visual analogue scales

The study was a double-blinded randomized parallel control trial examining the effect of 30 g/day inulin (Synergy1,Orafti, Tienen, Belgium) versus the control cellulose (Vitacel® Powdered Cellulose L 600–20, J. Rettenmaier & Söhne GmbH + Co.) alongside a 9-week weight loss and 9-week weight maintenance program (Fig. 1). The fibre cellulose was chosen as a comparator as this unbranched polymer of glucose molecules linked by (β4-1) bonds undergoes minimal colonic fermentation. The 30 g dose was chosen based on previous studies by our group [18, 19]. Both supplements were given as 10 g sachets taken three times a day with food or drink. To reduce potential gastrointestinal side-effects supplements were increased by 10 g/day every 2 weeks to reach the 30 g/day dose. Therefore, by week five of the nine-week weight loss phase, all subjects were on the maximum 30 g/day dose. The inulin and cellulose sachets were assigned letter A or B and were otherwise identical.

During the 9-week weight loss program each subject underwent a standardized dietary intervention comprising four dietary sessions 2–3 weeks apart with a registered dietitian (Fig. 1). The dietitian was blinded to the supplement allocation. Energy requirements and assigned portion sizes for starches, protein, milk and dairy, fats and sugars and fruits and vegetables were determined using a ready-reckoner developed at Hammersmith Hospital. Following the 9-week visit, subjects were given no further input or support from the study team. They were merely asked to try and maintain the weight that they had lost.

All subjects attended a study day at baseline, 9 weeks (representing the end of the weight loss phase) and 18 weeks (end of weight maintenance phase) for a meal tolerance test (MTT) (Ensure Plus™ (220 ml), Total energy: 1380 kJ, 44.4 g carbohydrate, 10.8 g fat, 13.8 g protein). Blood samples were taken at −15, 0, 5, 10, 15, 20, 30, 45, 60, 90, 120, 150, 180 min for measurement of glucose, insulin and GLP-1 (Fig. 1).

Appetite was assessed during the MTT using validated [20] visual analogue scales (VAS) at frequent intervals, followed by an *ad libitum* meal test [20]. The appetite questions used were “How hungry are you right now?” (general hunger), “How much do you think you could eat right now?” (prospective food consumption), “How pleasant would it be to eat right now?” (desire to eat) and “How full do you feel right now?” (feeling of fullness in the stomach) (Additional file 1).

Body composition was assessed by a 9-electrode bioelectrical impedance (BIA) device (Tanita BC-418 MA Segmental Body Composition Analyzer) (Tanita Corporation, Tokyo, Japan). The BIA device provides estimates of % body fat (±0.1 %), fat mass (±0.1 kg), and free fat mass (±0.1 kg). In addition, a subset of subjects who were eligible (no metal in situ or reported claustrophobia) (*n* = 20) underwent MRI and MRS to assess total and regional fat volumes at baseline, week nine and week 18. Rapid T1-weighted magnetic resonance images were obtained using a 1.5 T Phillips Achiva scanner (Phillips, Best, the Netherlands) [21] with intrahepatocellular lipid (IHCL), and intramyocellular lipid in the soleus and tibilalis muscles (IMCL-S and IMCL-T, respectively) assessed by MRS as previously described [22].

Compliance was assessed by asking subjects to return unused sachets and breath hydrogen was measured as a proxy of colonic fermentation using a breath hydrogen monitor (Gastrolyzer, Bedfont Scientific Ltd. Kent, UK).

### Laboratory analysis

Glucose was collected into fluoride oxalate tubes and measured using an Architect ci8200 analyzer (Abbott Diagnostics, Maidenhead, UK), with an assay detection limit of 0.3 mmol/L and an intra-assay coefficient of variation (CV) of 1 %. Plasma insulin samples were collected into serum gel separator tubes containing gel clotting activator Vacutte® and measured with a commercial radioimmunoassay kit (Millipore; Watford, UK). The sensitivity and intra-assay CV for insulin were 7.1 pmol, and 3.0 % respectively. GLP-1 samples were collected into lithium heparin tubes, with aprotinin (Trasylol, Bayer, Newbury, UK) (200 μL/7.5 ml blood) added. Blood samples were spun at 4000 g at 4 °C for 10 mins, separated into plasma and stored at −20 °C until analysed using an in-house radioimmunoassay [23]. The sensitivity and intra-assay CV of the GLP-1 assay were 7.5 pmol/l and 3.3 % respectively.

### Calculations and statistical analysis

Based on a study by Parnell et al. [24] with an expected weight loss of 2.0 kg and a standard deviation of 2.0 kg based on 0.8 power to detect a significant difference (*P* < 0.05, 2-sided) we estimated a minimum of 32 subjects were needed; 16 additional subjects were added to account for dropouts. Data are presented as mean ± standard error of the mean (SEM) for normally distributed data and median and interquartile range for non-normally distributed variables. The postprandial response curves for GLP-1, glucose and insulin were calculated as total area under the curve (tAUC) using the trapezoid rule. The primary outcome of weight change between the inulin and cellulose groups was calculated using an ANCOVA with baseline weight as a covariate. The delta change between the insulin and cellulose groups (between group difference) for glucose, inulin, GLP-1, and measures of adiposity and ectopic fat were compared using ANCOVAs, with change in weight as a covariate. Non-parametric tests were used for variables not normally distributed. A p value of less than 0.05 was considered significant. Analyses were performed using GraphPad Prism Version 5.0 (GraphPad Software, San Diego, CA) and ANCOVA was performed using SPSS 20.0 (SPSS Inc. Chicago, IL USA). The homeostatic model assessment of insulin resistance (HOMA-IR) and Matsuda index were used to measure fasting and postprandial insulin sensitivity [25, 26].

## Results

### Subject characteristics

The baseline characteristics of the subjects are shown in Table 1. There were no differences between the inulin and cellulose groups in any of the baseline characteristics. A total of 44 subjects were recruited and randomised of whom five withdrew from the study before week nine, including one taking inulin and one taking cellulose who dropped out due to side-effects. An additional subject withdrew following the week nine visit. See CONSORT diagram (Additional file 1). Furthermore, due to a drop-out (*n* = 1) scheduling difficulties (*n* = 2), withdrawal of consent for MRI (*n* = 1) and no longer being eligible for MRI (*n* = 1) five subjects who underwent baseline and week nine MRI scans did not have a scan at 18 weeks. Therefore 19 subjects completed a follow-up MRI at 9-weeks (inulin group: three females and seven males; cellulose group: four females and five males), and 14 subjects completed a follow-up MRI at 18 weeks insulin group: three females, six males; cellulose group: three females and two males).

**Table 1:** Subject characteristics for each arm of the study

	Inulin (*n* = 20)	Cellulose (*n* = 19)	*p* value
Gender (M:F)	13:8	11:9	0.66
Age (years)	58.2 ± 12.0	59.7 ± 8.9	0.53
Weight (kg)^b^	88.2 ± 14.0	83.4 ± 19.7	0.28
BMI (kg/m^2^)	30.8 ± 4.1	30.0 ± 2.3	0.41
FPG (mmol/L)	5.7 ± 0.5	5.9 ± 0.6	0.20
2hPG (mmol/L)	7.3 ± 2.3	7.3 ± 2.0	0.99
HbA1c (%) and (mmol/mol^a^)	5.9 ± 0.1 (41.3 ± 1.4)	5.7 ± 0.1 (38.8 ± 0.9)	0.78
Fasting insulin (pmol/L)	95.4 (116.8)^b^	114.1 (132.9)^b^	0.17

Values are means and SD. Gender is expressed as a ratio of males to females. There were no significant differences between the groups for any of the baseline variables. *FPG* fasting plasma glucose; *2hPG* 2-hour plasma glucose

^a^SI units for HbA1c shown in brackets

^b^Fasting insulin was not normally distributed so values given are for median (interquartile range)

### Compliance

On average, ten out of 189 sachets (5 %) were returned at week 18. The breath hydrogen levels increased by 15.0 ± 6.3 ppm (*n* = 20) in the inulin group vs 2.3 ± 1.6 ppm (*n* = 19) in the cellulose group at week nine (*p* = 0.07), a difference which reached significance at week 18 (14.8 ± 3.7 ppm, *n* = 20 vs 0.19 ± 0.8 ppm, *n* = 18, *p* < 0.001).

### Anthropological outcomes

#### Weight

**Fig. 2:**
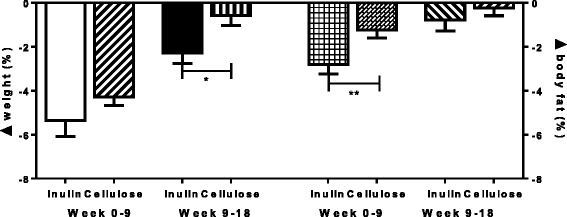
Percentage weight loss and body fat loss measured by BIA at week nine and week 18 in inulin and cellulose groups. Analysis was done by ANCOVA with baseline weight as a covariate. Weight loss at week nine was not significantly different between inulin and cellulose groups (−5.3 ± 0.1 %, *n* = 20 vs −4.3 ± 0.4 %, *n* = 19, *p* = 0.13). Between weeks 9–18 the inulin group lost significantly more than the cellulose group (−2.3 ± 0.5 %, *n* = 20 vs −0.6 ± 0.4 %, *n* = 18, *p* = 0.012). Analysis for body loss was done by ANCOVA with baseline weight as a covariate. The inulin group lost a greater percentage of body fat as measured by BIA at 9 (−2.8 ± 0.4 %, *n* = 20 vs −1.2 ± 0.4 %, *n* = 19, *p* < 0.01) and 18 weeks (−3.7 ± 0.6 %, *n* = 20 vs −1.1 ± 0.6 %, *n* = 18, *p* = 0.01). ANCOVA: analysis of covariance; BIA: bioelectrical impedance

As intended by the study design, both groups lost similar amounts of weight during the weight loss phase (−5.3 ± 0.1 %, *n* = 20 vs −4.3 ± 0.4 %, *n* = 19, *p* = 0.13). Measured as absolute weight loss: −4.6 kg ± 0.6 kg vs −3.61 ± 0.3 kg. However, during the weight maintenance phase (weeks 9–18), the inulin group lost significantly more weight compared to the control (−2.3 ± 0.5 %, *n* = 20 vs −0.6 ± 0.4 %, *n* = 18, *p* = 0.012). Measured as absolute weight loss: −1.8 ± 0.4 kg vs −0.5 ± 0.3 kg (Fig. 2).

#### Body composition

Body fat percent as analysed by BIA at week 9 and 18 showed a greater reduction in the inulin group compared to the cellulose group (−2.8 ± 0.4 %, *n* = 20 vs −1.2 ± 0.4 %, *n* = 19, *p* < 0.01) and (−3.7 ± 0.6 %, *n* = 20 vs −1.1 ± 0.6 %, *n* = 18, *p* = 0.01) respectively (Fig. 2). In the sub-group of subjects who underwent MRI, the percentage body fat measurements at 9-weeks showed a similar trend towards a reduction in body fat (−1.7 ± 0.6 %, *n* = 10 vs −0.1 ± 0.6 %, *n* = 9, *p* = 0.08) although this did not reach statistical significance. The delta change was not different at 18 weeks (−2.4 ± 1.2 %, *n* = 9 vs −2.2 ± 3.1 %, *n* = 5, *p* = 0.93). The delta change in other fat depots was not different at 9 or 18 weeks (Additional file 1).

### Intrahepato- and intramyocellular lipid

**Fig. 3:**
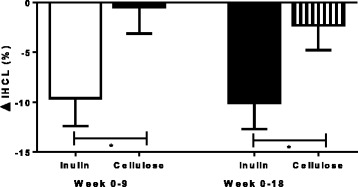
Change in intrahepatocellular lipid (IHCL) at weeks 9 and 18 in inulin and cellulose groups. Analysis was done by ANCOVA with change in body weight as a covariate. IHCL was significantly reduced in subjects randomised to the inulin supplement compared to the cellulose at 9 (−9.6 ± 2.8 %, *n* = 10 vs −0.5 ± 2.7 %, *n* = 9, *p* < 0.04) and 18 weeks (−10.0 ± 2.6 %, *n* = 9 vs −2.3 ± 2.5 %, *n* = 5, *p* = 0.02). ANCOVA: analysis of covariance; IHCL: intrahepatocellular lipid

**Fig. 4:**
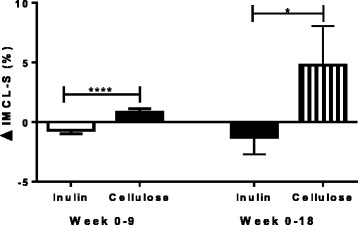
Change in intramyocellular lipid in the soleus muscle (IMCL-S) at weeks 9 and 18 in inulin and cellulose groups. Analysis was done by ANCOVA with change in body weight as a covariate. IMCL-S was significantly reduced at 9 and 18 weeks in the inulin group compared to cellulose: (9 weeks: −0.7 ± 0.3 %, *n* = 10 vs 0.8 ± 0.3 %, *n* = 9, *p* < 0.005); (18 weeks: −1.3 ± 1.4 %, *n* = 9 vs 4.8 ± 3.0 %, *n* = 5, *p* < 0.05). ANCOVA: analysis of covariance; IHCL: intrahepatocellular lipid

Intrahepatocellular lipid was reduced in the inulin group, and the delta change was significant when compared to the cellulose group at 9 and 18 weeks even after controlling for weight loss (9 weeks: −9.6 ± 2.8 %, *n* = 10 vs −0.5 ± 2.7 %, *n* = 9, *p* < 0.04); (18 weeks: −10.0 ± 2.6 %, *n* = 9 vs −2.3 ± 2.5 %, *n* = 5, *p* = 0.02) (Fig. 3). The fat content of the soleus muscle was also significantly reduced at 9 and 18 weeks (9 weeks: −0.7 ± 0.3 %, *n* = 10 vs 0.8 ± 0.3 %, *n* = 9, *p* < 0.005); (18 weeks: −1.3 ± 1.4 %, *n* = 9 vs 4.8 ± 3.0 %, *n* = 5, *p* < 0.05) (Fig. 4) but not the tibialis muscle (9 weeks: −1.4 ± 1.0 % *n* = 10 vs −1.0 ± 1.4 %, *n* = 9, *p* = 0.82); (18 weeks: −2.2 ± 0.8 %, *n* = 9 vs −2.5 ± 2.7 %, *n* = 5, *p* = 0.93).

### Measures of appetite

#### Visual analogue scales

Subjective appetite assessment showed no differences in tAUC hunger, desire to eat, or fullness at 9 and 18 weeks between the inulin and cellulose groups (Additional file 1). There was no difference in tAUC for the question “How much do you think you could eat right now?” (prospective food consumption) at week nine (−62.9 ± 58.2, *n* = 20 vs −117.8 ± 44.2, *n* = 19, *p* = 0.46) but subjects in the cellulose reported significantly greater tAUC for the prospective food consumption question than the inulin group at week 18 (4.5 ± 37.9, *n* = 20 vs 774.5 ± 86.97, *n* = 18) *p* = <0.0001).

#### Food intake from *Ad Libitum* meal

Subjects in the inulin group ate significantly less than the cellulose group at week nine compared to the baseline visit (−127.0 ± 45.4 g, *n* = 20 vs −0.47 ± 22.5 g, *n* = 19, *p* = 0.027). There were no differences in food intake between baseline and week 18 between the inulin and cellulose groups (−87.3 ± 51.3 g, *n* = 20, vs −1.2 ± 23.7 g, *n* = 18, *p* = 0.18) (Additional file 1).

### Biochemical outcomes

#### Glucose

**Fig. 5:**
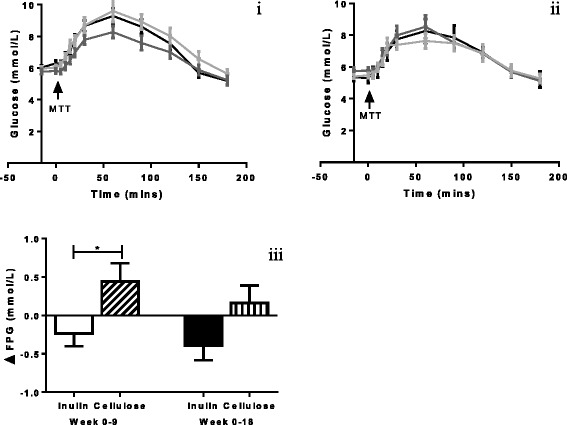
Time course data for plasma glucose at baseline, 9-weeks and 18 weeks for inulin (**i**) and cellulose supplementation (**ii**). Black line = baseline; dark grey line = 9-week visit; light grey line = 18-week visit. tAUC was calculated using the trapezoid method. ANCOVA using weight change as a covariate was used to analyse delta change between the inulin and cellulose groups. Glucose tAUC delta change at week nine(135 ± 134, *n* = 17 vs 87 ± 42, *n* = 17, *p* = 0.37) or at week 18 (−267 ± 104, *n* = 17 vs −38 ± 21, *n* = 17, *p* = 0.37) did not differ between groups once controlled for weight loss. There was a significant difference in the FPG delta change between groups at week nine once controlled for weight loss (−0.23 ± 0.17 mmol/L, *n* = 20 vs 0.44 ± 0.24 mmol/L, *n* = 19, *p* = 0.005) while the change at week 18 was no longer significant (−0.40 ± 0.19 mmol/L, *n* = 20 vs 0.16 ± 0.23 mmol/L, *n* = 18, *p* = 0.08) (iii). ANCOVA: analysis of covariance; FPG; fasting plasma glucose; tAUC: total area under the curve

There was a significant reduction in fasting plasma glucose (FPG) in the inulin group compared to control at week nine (−0.23 ± 0.17 mmol/L, *n* = 20 vs 0.44 ± 0.24 mmol/L, *n* = 19, *p* = 0.005) after controlling for weight loss but the change at week 18 was no longer significant (−0.40 ± 0.19 mmol/L, *n* = 20 vs 0.16 ± 0.23 mmol/L, *n* = 18, *p* = 0.08) (Fig. 5i and ii). Although glucose tAUC decreased in both groups, there was no between group difference at week nine (*p* = 0.37) or at week 18 (*p* = 0.37) once controlled for weight loss (Fig. 5iii).

#### Insulin

**Fig. 6:**
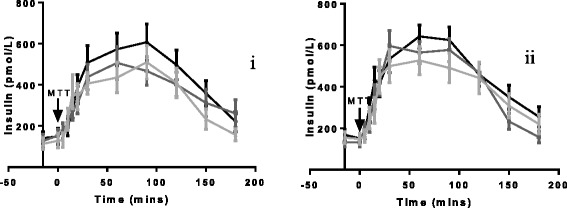
Time course data for plasma insulin at baseline, 9 weeks and 18 weeks for inulin (**i**) and cellulose supplementation (**ii**). Black line = baseline; dark grey line = 9-week visit; light grey line. tAUC was calculated using the trapezoid method. ANCOVA using weight change as a covariate was used to analyse delta change between the inulin and cellulose groups. After controlling for weight lost the delta change in insulin tAUC following inulin supplementation was similar to the cellulose group at week nine (−2366 ± 575, *n* = 17 vs −1566 ± 1724, *n* = 17, *p* = 0.66) and week 18 (−2643 ± 671, *n* = 17 vs −1264 ± 1045, *n* = 17, *p* = 0.27). The delta change in fasting insulin was also similar between inulin and cellulose at week nine (−23.3 ± 15.4 pmol/L, *n* = 18 vs −30.5 ± 17.6 pmol/L, *n* = 17, *p* = 0.82) and week 18 (−35.5 ± 17.8 pmol/L, *n* = 18 vs −14.7 ± 18.7 pmol/L, *n* = 17, *p* = 0.53). ANCOVA: analysis of covariance; tAUC: total area under the curve

As expected following weight loss, the insulin tAUC decreased in both inulin and cellulose groups (Fig. 6i and ii), but there were no between-group differences at week nine (*p* = 0.66) or week 18 (*p* = 0.27). The delta change in fasting insulin was also similar between inulin and cellulose at week nine (−23.3 ± 15.4 pmol/L, *n* = 18 vs −30.5 ± 17.6 pmol/L, *n* = 17, *p* = 0.82) and week 18 (−35.5 ± 17.8 pmol/L, *n* = 18 vs −14.7 ± 18.7 pmol/L, *n* = 17, *p* = 0.53). Note, due to haemolysis it was not possible to analyse all samples.

#### Insulin sensitivity

There were no differences in delta change of HOMA-IR at week nine (−0.6 ± 0.3, *n* = 18 vs −0.4 ± 0.3, *n* = 17, *p* = 0.65) and week 18 (−0.7 ± 0.3, *n* = 18 vs −0.2 ± 0.4, *n* = 17, *p* = 0.23) or Matsuda Index at week nine (0.5 ± 0.5, *n* = 17 vs 0.01 ± 0.2, *n* = 17, *p* = 0.56) or week 18 (0.4 ± 0.7 *n* = 17, vs 0.1 ± 0.3, *n* = 17, *p* = 0.75) between the inulin and cellulose groups after adjusting for weight lost.

### GLP-1

At 9 and 18 weeks the GLP-1 tAUC decreased following inulin supplementation and increased in the cellulose group and the delta changes between the groups were significantly different (−1005 ± 457, *n* = 17 vs 614 ± 399, *n* = 16, *p* < 0.03) and 18 weeks (−525 ± 315, *n* = 17 vs 4500 ± 710.4 *n* = 16, *p* < 0.0001), respectively.

## Discussion

Weight loss reduces the risk of diabetes, and medications which reduce appetite can promote long-term weight loss [7]. Previous studies have shown that FCHO reduces appetite, food intake and body weight, independent of lifestyle change [10, 18, 24]. We extend these findings to show that the consumption of inulin enhances a traditional calorie-restricted lifestyle program. An added benefit of the inulin supplement was a greater reduction in intrahepatocellular and intramyocellular lipid in the soleus muscle even after accounting for weight lost.

Expert bodies recognize the importance of frequent visits in primary care (more than once a month, face-to-face contact during the first three months) in achieving long-term weight loss maintenance [27]. However, the labour-intensive nature of this approach reduces its widespread applicability. Therefore an important component of our study design was to ensure that the subjects would not receive dietary advice or support during the final 2 months of the study. Our finding that the inulin group lost more weight between weeks 9–18 compared to cellulose therefore suggests that inulin may be a useful adjunct to traditional lifestyle approaches to diabetes prevention. Furthermore, despite the 5 % weight loss at week nine, subjects taking inulin ate significantly less (~270 kcal less, *p* = 0.027) at the follow-up *ad libitum* meal, with no consequent rebound in food intake at the 18 week visit despite a total 7 % weight loss in the inulin group, suggesting that inulin’s effect on weight management is mediated via appetite modulation.

Unexpectedly, the cellulose group also continued to lose weight during the weight maintenance period, albeit a modest amount. Firstly, the length of the study was likely not long-enough to measure true long-term weight loss maintenance, and both groups may have simply continued with the dietary changes they had made during the 9-week weight loss period. Weight loss tends to plateau at 6 months, after which weight exhibits a gradual but continuous rise [28]. We had selected the 9-week weight loss and 9-week weight maintenance periods as a balance between degree of participant burden and feasibility [29, 30]. Our findings at this time therefore support a role for inulin in weight loss, as opposed to weight-loss maintenance. We suggest that a longer follow-up period should be incorporated in future study designs.

Secondly, we had selected cellulose as the control as it is known to be the least fermentable of the fibres [31]. However, adaptations of the gut microbiota over time have also been reported following cellulose ingestion [32] and cereal fibre (24 g per day) has been shown to increase GLP-1 secretion taken over a year [33]. We suggest that more work should be carried out to understand the physiochemical properties of dietary fibres and their relationship to weight management. Nevertheless, since dietary fibre is inversely associated with BMI [8, 34] the use of cellulose as a control group would suggest our data underestimate the likely magnitude of effect of inulin on weight loss.

Inulin consumption not only promoted weight loss but was also associated with greater loss of triglyceride in the liver (IHCL) and soleus muscle (IMCL-S), even after controlling for differences in weight lost. We were able to collect MRI and MRS data on 19 subjects, and similar studies examining, IHCL, IMCL and adiposity have used comparable sample sizes [35, 36]. We therefore extend findings of a beneficial effect of FCHO on ectopic fat from animal studies [14], and from human studies in non-alcoholic fatty liver disease [37] to suggest that FCHO is also able to reduce IHCL and IMCL-S content in subjects at risk of diabetes independent of weight loss. A previous study has shown that 12 weeks’ supplementation with the FCHO oligofructose led to greater weight loss as fat as measured by x-ray absorptiometry [24]. However, our data on the proportion of weight lost as fat are inconclusive, given the lack of significance in the MRI data and the caution required in interpreting bioimpedance measurements.

The reduction in IHCL following inulin supplementation is of particular importance given the potent and consistent relationship between triglyceride content of the liver and metabolic disease, particularly diabetes [11, 13]. Furthermore, previous studies have found an increase in lipid content of the soleus muscle but not the tibialis in the off-spring of subjects with type II diabetes [12] and Indian males with diabetes have significantly higher IMCL-content in the soleus compared to healthy controls [38]. The greater oxidative muscle fibre content of the soleus muscle may be related to insulin resistance due to altered insulin-dependent glucose transport [39].

The mechanism by which FCHO reduces fat deposition appears to be related to increased fat oxidation. Rats fed a diet high in viscous (fermentable) fibres have increased expression of carnitine palmitoyltransferase 1B (CPT-1B) – the rate-limiting enzyme in the soleus muscle, alongside other key fat oxidation genes: peroxisome proliferator-activated receptors 1α and δ, uncoupling protein 3, and citrate synthase [40]. In humans, fat oxidation is increased 3 h after consumption of a low glycaemic index breakfast, indicating that fermentation of unavailable carbohydrates may influence oxidation in vivo [41]. It has also recently been shown that inulin down-regulates hepatic lipid production alongside increases in portal SCFA concentrations in mice fed a high-fat diet [42].

Despite the effect of inulin on liver fat we did not observe any added effect on insulin sensitivity after accounting for weight loss. Dietary studies on the effects of FCHO on insulin sensitivity have shown improvements in peripheral and/or hepatic insulin sensitivity [15, 16, 43]. We did not find any changes in fasting (HOMA-IR) or postprandial insulin sensitivity (Matsuda Index) in this study. However, the distinct pathophysiology which underpins the different prediabetic states may have been a confounding factor. Subjects with IFG are known to have hepatic insulin resistance, but normal peripheral insulin sensitivity [44]; while subjects with IGT are known to have normal or mild hepatic, but marked peripheral insulin resistance [44]. It is not known whether inulin affects hepatic or peripheral insulin sensitivity, and this study was not powered to examine differences between the prediabetic states. This should be considered in future studies. Nevertheless, there was a significant reduction in FPG after controlling for weight loss in the inulin group at week nine. It is unclear why this was no longer different at week 18, but insoluble fibres have shown repeated inverse associations with diabetes risk in cohort studies [45], with the mechanism currently unknown. Potentially, the fermentation of cellulose and the rise in GLP-1 may have played a role. Furthermore, other changes made in the diet in both the inulin and cellulose groups may also have contributed, as all subjects were encouraged to eat five or more servings of fruit and vegetables daily and increase their consumption of high-fibre foods. Total dietary fibre is known to be inversely related to a number of cardiovascular risk factors [46]; however our data point to specific metabolic effects of fermentable fibres which may be of particular benefit to individuals at risk of diabetes. Use of stool analysis in future studies would be very useful to help delineate some of these effects.

We acknowledge some limitations in the study. While compliance in dietary studies is always challenging, the breath hydrogen significantly increased when the supplement was being taken, providing a degree of dietary compliance. The high return rate of the used sachets also suggests that the effects seen in this study were due to chronic consumption of the supplementation. We also did not measure physical activity which is known to affect insulin sensitivity and loss of ectopic fat. We therefore cannot rule out that exercise may have influenced the results seen here, and activity meters should be used in future studies. Finally, there are known gender differences in body composition [47]. While the study did not aim to determine gender differences in the study outcomes, this is certainly something that should be examined in a study powered to do so.

## Conclusion

In conclusion, our findings support a role for fermentable fibres in modifying two key risk factors in diabetes development: weight management and ectopic fat deposition. Inulin, and possibly other fermentable carbohydrates, could therefore represent a potentially effective intervention at the population level.

## Additional file

Additional file 1: **Supplementary material.** (DOCX 87 kb)

## Abbreviations

ANCOVA: Analysis of covariance
BIA: Bioelectrical impedance
CPT-1b: Carnitine palmitoyltransferase 1B
CV: Coefficient of variation
FCHO: Fermentable carbohydrate
FPG: Fasting plasma glucose
GLP-1: Glucagon-like peptide 1
HOMA-IR: Homeostasis model assessment of insulin resistance
IFG: Impaired fasting glucose
IGT: Impaired glucose tolerance
IHCL: Intrahepatocellular lipid
IMCL: Intramyocellular lipid
MRI: Magnetic resonance imaging
MRS: Magnetic resonance spectroscopy
MTT: Meal tolerance test
OGTT: Oral glucose tolerance test
SCFA: Short-chain fatty acids
SEM: Standard error of the mean
tAUC: Total area under the curve
T2DM: Type 2 diabetes
VAS: Visual analogue scales
VAT: Visceral adipose tissue
2hPG: 2 h plasma glucose
