# Supplementary material for: A randomized controlled trial: the effect of inulin on weight management and ectopic fat in subjects with prediabetes
Source: Nutr Metab (Lond). 2015 Oct 24;12:36. doi: 10.1186/s12986-015-0033-2 (PMC4619305; doi:10.1186/s12986-015-0033-2)
Supplement: Supplementary file 1 — Supplementary material. (DOCX 87 kb) [file 12986_2015_33_MOESM1_ESM.docx]

**Additional file 1**

**Visual Analogue Scales Questions used:**

How hungry are you right now?

Not at all Very

How pleasant would it be to eat right now?

Not at all Very

How much do you think you could eat right now?

Nothing at all A lot

How full do you feel right now?

Not at all A lot

**CONSORT Flow Diagram**

Telephone screened for eligibility (n=421)

Excluded (n=137)

♦  Not meeting inclusion criteria (n=122)

♦  Declined to participate (n=15)

♦  Other reasons (n=4)

Assessed for eligibility (n=181)

## Enrollment

Randomized (n=44)

## Allocation

Allocated to inulin (n=22)

Received allocated intervention (n=22)

Allocated to cellulose (n=22)

Received allocated intervention (n=22)

Discontinued intervention in weight loss phase (n=1; side effects n=1; time constraints n=1)

## Follow-Up

Discontinued intervention in weight loss phase (side effects n=1; medical reasons n=2). Discontinued intervention in weight maintenance (time constraints; n=1)

Analysed at week 9 (n=19)

Analysed at week 18 (n=18)

Analysed at week 9 (n=20)

Analysed at week 18 (n=20)

## Analysis

COHORT diagram showing flow of participants through recruitment, screening and end of study.

| **Fat Depot** | **Group** | **Pre (%)** | **Post (%)** | **Difference (%)** | **P Value (a)** | **P Value (b)** |
| --- | --- | --- | --- | --- | --- | --- |
| Sc-AT | Inulin | 28.69 ± 2.80 | 27.50 ± 3.07 | -1.20 ± 4.16 | 0.0094 | 0.0837 |
|  | Cellulose | 28.00 ± 2.95 | 27.60 ± 2.92 | -0.40 ± 4.16 | 0.1550 |  |
| Int-AT | Inulin | 8.85 ± 0.68 | 8.12 ± 0.78 | -0.73 ± 1.04 | 0.0920 | 0.1089 |
|  | Cellulose | 8.14 ± 1.05 | 8.26 ± 1.00 | +0.11 ± 1.45 | 0.7225 |  |
| Sc_AAT | Inulin | 8.62 ± 0.90 | 8.15 ± 0.98 | -0.47 ± 1.3 | 0.0136 | 0.2572 |
|  | Cellulose | 8.02 ± 0.72 | 7.76 ± 0.77 | -0.25 ± 1.05 | 0.0495 |  |
| P-AAT | Inulin | 20.07 + 2.08 | 19.34 + 2.27 | -0.72 ± 3.08 | 0.0225 | 0.1329 |
|  | Cellulose | 19.98 ± 2.32 | 19.84 ± 2.20 | -0.14 ±3.20 | 0.6005 |  |
| VAT | Inulin | 5.10 ± 0.38 | 4.57 ± 0.44 | -0.53 ± 0.58 | 0.0457 | 0.1753 |
|  | Cellulose | 4.823 ± 0.80 | 4.73 ± 0.73 | -0.09 ± 1.09 | 0.6680 |  |
| Non-VAT | Inulin | 3.74 ± 0.33 | 3.55 ± 0.37 | -0.20 ± 0.50 | 0.3364 | 0.1031 |
|  | Cellulose | 3.32 ± 0.30 | 3.53 ± 0.32 | +0.21 ±0.445 | 0.1617 |  |

Fat content pre- and post- inulin and cellulose supplementation in regional fat depots. a) Paired t-test of within group difference. b) Independent t-test for between group difference. Sc-AT: Subcutaneous adipose tissue; Int-AT: Internal adipose tissue; Sc-AAT: subcutaneous abdominal adipose tissue; P-AAT: Peripheral Abdominal adipose tissue; VAT – Visceral adipose tissue.

|  | Inulin | | | Cellulose | | |
| --- | --- | --- | --- | --- | --- | --- |
|  | Pre | Post | Difference | Pre | Post | Difference |
| Hunger  (9 weeks) | 810 ± 66 | 745 ± 51 | -64 ± 83 | \| 610 ± 87 \| \| --- \| | 682 ± 78 | \| 91 ± 112 \| \| --- \| |
| Hunger  (18 weeks) | 745 ± 51 | 774 ± 91 | 28 ± 105 | 727 ± 71 | 861 ± 77 | 134 ± 105 |
| Pleasant  (9 weeks) | 950 ± 79 | 855 ± 75 | -95 ± 109 | 873 ± 85 | 777 ± 109 | -96 ± 138 |
| Pleasant  (18 weeks) | 855 ± 75 | 794 ± 100 | -61 ± 125 | 826 ± 107 | 822 ± 116 | -4 ± 158 |
| Prospective  (9 weeks) | 884 ± 63 | 767 ± 57 | -116 ± 85 | 833 ± 69 | 682 ± 71 | -151 ± 100 |
| Prospective  (18 weeks) | 767 ± 57 | 768 ± 86 | 0.8 ± 104 | 706 ± 74 | 807 ± 74 | 100 ± 105^a^ |
| Full  (9 weeks) | 569 ± 76 | 548 ± 42 | -20 ± 87 | 636 ± 93 | 731 ± 101 | 95 ± 138 |
| Full  (18 weeks) | 548 ± 42 | 578 ± 73 | 29 ± 84 | 691.5 ± 102.0 | 610 ± 87 | -81 ± 134 |

Table showing the pre- and post tAUC for both inulin and cellulose groups for the visual analogue scales (VAS) used. There were no differences in tAUC hunger (Week 9: -74.9 ± 66.3 vs -85.7 ± 49.1, P = 0.8993; (Week 18: 70.2 ± 64.7 vs 2.8 ± 77.8, P = 0.5129) tAUC pleasant (Week 9: -74.9 ± 56.5 vs -159.4 ± 69.9, P = 0.3639; Week 18: -56.1 ± 37.7 vs -38.7 ± 53.8, P = 0.7907), or tAUC full (Week 9: 28.1 ± 38.0 vs 44.6 ± 48.8, P = 0.7896; Week 18: (14.2 ± 45.8 vs -87.0 ± 45.0, P = 0.1266) between the inulin and cellulose groups. There was no difference in tAUC for prospective food consumption at week 9 (-62.9 ± 58.2 vs -117.8 ± 44.2, P =0.4614) but subjects in the cellulose reported significantly greater tAUC for prospective food consumption than the inulin group at week 18 (4.5 ± 37.9 vs 774.5 ± 86.97, P = <0.0001).

Delta change in food intake at the *ad libitum* meal following inulin or cellulose supplementation between weeks 0-9 and weeks 0-18. Subjects in the inulin group ate significantly less than the cellulose group at week 9 compared to the baseline visit (-127.0 ± 45.4g, n=20 vs -0.47 ± 22.5g, n=18, p=0.027). There were no differences in food intake between baseline and week 18 between the inulin and cellulose groups (-87.3 ± 51.3g, n=20, vs -1.2 ± 23.7g, n=17, p=0.18).
